# Mr. Creaser, on Spurious Vaccine

**Published:** 1801-10

**Authors:** T. Creaser

**Affiliations:** Surgeon. Bath


### *To the Editors of the Medical and Phyfical Journal.*

GENTLEMEN,

AN Enquirer in your laft month's Journal, under the fignature of M. propofes a queftion, the folution of which may not only be fatisfactory to himfelf, but is explanatory of the moft important principle in the conduct of Vaccine Inoculation. *M.* enquires, "Whether the virus, taken after the full formation of the areola, never produces the genuine Cow-pox, or if it only fometimes fails; and, in this cafe, by what characteriftic the imitative difeafe is to be diftinguifhed from the genuine?"

Intimate acquaintance with the opinions of Dr. Jenner, and a confiderable fhare of experience in Vaccine Inoculation, induce me to attempt a reply to this query. There is not a fact in the hiftory of Cow-pox better afcertained, than that the virus, at a late period, undergoes a chemical change from the altered living action of the part which fecretes it; and that in this new combination, its fpecific properties are altered, and others are affumed. This altered fecretion is capable of producing morbid and phagedenic ulceration, confiderable eryfipelatous inflammation, and a train of effects wholly diffimilar to thofe of pure and recently formed virus. The precife and definitive definitive time at which this change in the conftituent qualities of the virus occurs, is probably different in different fubjects; but in the formation of a general rule, it is neceffary to impofe a limitation which will be conftantly correct. This point will be attained by never employing virus of a later exiftence than the eighth or ninth day, a period previous to which no alteration in its qualities can occur, provided the progreffion of the vaccine puftule has been duly regular. But if virus of a later date happens to be employed, it certainly *may* produce the perfect and genuine difeafe, which will be recognifed by the appearances which have been fo fully and fo accurately defined.

I am, &c.

*Bath, Sept.* 16, 1801. T. CREASER, Surgeon.

